# GMFG (glia maturation factor gamma) inhibits lung cancer growth by activating p53 signaling pathway

**DOI:** 10.1080/21655979.2022.2049958

**Published:** 2022-04-06

**Authors:** Hua Tang, Jie Liu, Jun Huang

**Affiliations:** a Department of Thoracic Surgery, Shanghai Changzheng Hospital, Navy Military Medical University, Shanghai, Shanghai, China; b Department of Thoracic Surgery, Army medical university, Southwest hospital, Chongqing, Sichuan , China; c Department of Thoracic Oncology, The First Affiliated Hospital of Guangzhou Medical University, Guangzhou, Guangdong, China

**Keywords:** Proliferation, p53, lung cancer, GMFG

## Abstract

The tumor-promoting or tumor-suppressing functions of Glia maturation factor gamma (GMFG) were described in several cancers. However, how GMFG regulates lung cancer progression is elusive. Bioinformatics analysis was employed to analyze GMFG expression in lung adenocarcinoma (LUAD) and lung squamous cancer (LUSC) as well as its significance in prognosis prediction and diagnosis in lung cancer patients. CCK8 and colony formation assays were adopted to evaluate the impact of GMFG overexpressing and depleting on lung cancer cell proliferation. And in vivo experiments were implemented. Luciferase reporter assays were used to disclose the signaling pathway mediated by GMFG in lung cancer. GMFG expression was lower in LUSC and LUAD tissues compared with normal lung tissues based on TCGA and GTEx databases. Low GMFG expression was associated with lower overall survival and shorter disease specific survival compared high GMFG expression. In vitro loss and gain functions assays demonstrated that ectopically GMFG expression dampened the lung cancer cell proliferation while GMFG knockout escalated the cell proliferation. The promoting effect of GMFG knockout on lung cancer tumorgenesis was also observed in vivo. More interesting, GMFG overexpression reinforced the p53 signaling pathway in lung cancer cells, conversely GMFG deficiency disrupted p53 signaling pathway. In conclusion, we revealed that GMFG is fundamental to p53 signaling pathway to inhibit lung cancer progression, highlighting the importance of GMFG as a p53 inducer for lung cancer patient’s diagnosis and therapy.

## Introduction

Lung cancer is the second frequently diagnosed malignancy following breast cancer, accounting for 11.4% of all newly-diagnosed cases [1]. An approximately 18% fatality rate was recorded in individuals suffering from lung cancer. Among lung cancer, about 85% are non-small cell lung cancer (NSCLC) which are further histologically subgrouped into lung adenocarcinoma (LUAD) and lung squamous cancer (LUSC) [2]. The current mainstay interventions against lung cancer are mostly beneficial for the patients in early stage [3]. However, most cases are found at advanced stages due to inconspicuous symptoms. Therefore, better deciphering the underlying mechanism is instrumental to lung cancer diagnosis and therapy.

Glia maturation factor gamma (GMFG) gene locates on chromosome 19q13.2 and consists of 7 exons. It encodes a member of actin-depolymerizing factor (ADF)/cofilin family which is critical for reshaping the actin cytoskeleton. Originally, GMFG protein is assumed as a glia maturation factor which is a critical inducer of brain cell growth [4]. However, currently accumulating evidence showed that GMFG is involved in the initiation of daughter filament growth, enabling neutrophil and T cell migration [5]. Due to the important role of the actin cytoskeleton remodeling in cancer cell fate, angiogenesis [6] and tumor immune evasion [7], cellular iron metabolism [8], its biofunction in various cancer interested researchers. Zou et al demonstrated that GMFG is highly expressed in epithelial ovarian cancer and negatively associated with clinical outcome [9]. Furthermore, the lessened migratory and invasive capacities of colorectal cancer cells is a consequence of GMFG silence [10]. However in breast cancer, GMFG is found to be poorly expressed in cancer tissues, dismal clinical outcome are detected in breast patients with low GMFG [11]. The context-dependent expression of GMFG was also validated by a TCGA pan-cancer analysis which reveals a low expression of GMFG in LUAD and LUSC [12]. However, no investigation is focused on the underlying mechanism in lung cancer.

In our present work, we aimed to explore the role of GMFG during lung cancer progression. Firstly, we verified GMFG expression and forecasted its prognostic and diagnostic values using TCGA and GTEx database. Following, we applied loss and gain functional assays to validate whether the GMFG is necessary for lung cancer progression. Meanwhile, a group of luciferase reporter vectors were applied to screen the potential signaling pathway. Consequently, this study offers molecular mechanistic insight into how GMFG suppresses the lung cancer growth, proposing targeting GMFG might be a druggable approach to combat lung cancer.

## Methods

### Bioinformatics analysis

TCGA-LUAD dataset and TCGA-LUSC database were obtained from TCGA data portal (https://tcga-data.nci.nih.gov/tcga) [13]. The GMFG expression was also acquired from GTEx database (https://www.gtexportal.org/home/). Based on these TCGA database and GTEx database [14], we applied the ggplot2 package to visualize the differential expression of GMFG mRNA in TCGA cancer tissues and TCGA normal tissues as well as that in TCGA cancer tissues and TCGA+GTEx normal tissues. The survival and ROC curves were plotted using ggplot2 packages

### Cell culture and plasmids

Human lung cancer cell lines A549 and H1395 cells were purchased from Cell Storage Center of Wuhan University (Wuhan, China). A549 cells (F-12 K medium) and H1395 cells (RPMI-1640 medium) were maintained in the corresponding medium containing 10% fetal bovine serum and 1% penicillin/streptomycin at a 37°C chamber with 5% CO_2_.

ATF6 Luciferase reporter plasmids, p53 luciferase reporter plasmids, E2F luciferase reporter plasmids, GR luciferase reporter plasmids, GLI luciferase reporter plasmids, AP1-luc Reporter gene plasmids and Myc luciferase reporter plasmids were purchased from Yeason Biological Technology Co. Ltd, Shanghai, China.

### Construction of GMFG-overexpressing lung cancer cells

For GMFG ectopic expression, the recombinant vectors pcDNA3.1-Flag-GMFG were established by Tianyi Huayu, Biotech Co.Ltd.,Wuhan, China. A549 and H1395 cells at 75% confluence were transfected with pcDNA3.1-Flag-GMFG and the empty vectors with the help of Lipofectamine 3000 (ThermoFisher, USA) in line of the manufacture’s instruction. 48 h posttransfection, GMFG overexpression were verified by western blots with Flag antibodies.

### Construction of GMFG-deficient lung cancer cells

For GMFG knockout, sgRNA recognizing GMFG exon 1 and sgRNA target none were designed through the http://crispr.mit.edu [15] and their synthesized oligonucleotides were inserted into LentiCRISPRv2 vectors. The produced LentiCRISPRv2-sgRNA-GMFG exon 1 constructs or the control vectors were introduced into 293 T cells using Lipofectamine 3000. After 48 h, the medium containing lentiviral particles were subjected to high-speed centrifugation and viral titer estimation using high-performance liquid chromatography. A virus titer of >1 × 10^8^ infectious particles/ml was infected into A549 and H1399 cells. 48 h posttransfection, 2 µg/ml puromycin were used to screen the cells. After 2 weeks, the positive clones were collected and propagated, following verification with western blots and Sanger sequencing.

### RT-qPCR

Total RNA was extracted from A549 and H1395 cells using TRIzol reagent. 2 μg RNAs were reverse transcribed into cDNA using a ReverTra Ace qPCR RT Kit (Toyobo life science, Japan). The mRNA of Bax, p21 and GMFG were evaluated by qPCR on 7500 Real Time PCR system (Applied Biosystems). 2^−ΔΔCT^ [16] method was utilized to analyze the relative expression of target genes with normalization with GAPDH. p21 forward primer 5′-GCCAGATTTGTGGCTCACTTCG and p21 reverse primer 5′-ACGCTTGGCTCGGCTCTGG; BAX Forward primer-TGAAGACAGGGGCCTTTTTG; Reverse primer-AATTCGCCGGAGACACTCG;

### Western blot

Cells and mice lung tissues were treated with RIPA buffer (Amylet Scientific, China). After microcentriguation, the protein concentration was determined by Bio-Rad protein assay dye reagent (Biorad, Australia). 20 ug prepared protein samples were electrophoresed in 10% SCD-PAGE at constant 80 Volts and then electrotransferred into PVDF membranes. The diluted primary antibodies were added and incubated with the membranes at 4°C after the membranes blocking using 5% skim milk powder. Next day, the membranes were exposed to the secondary antibodies at room temperature for 1 h. ECL was used to developed blots with X-ray films.

### CCK8 assays

Cells in logarithmic phase (1 × 10^3^ cells/well) were maintained in 96-well plates. The plates were read with a microtiter plate reader at OD450 nm every 2 days after 10 µl CCK8 solution (ThermoFisher, USA) was supplemented into each well for 3 h [17].

### Colony formation assay

3 x 10^4^ cells in logarithmic phase were seed in 6-well plates. After 10-day incubation at 37°C with 5% CO_2_, cells were subjected to 15-min fixation with 4% paraformaldehyde for 15-min following staining with GIMSA solution (Thermofisher, USA) for 30 min. The number of colonies more than 50 cells were macroscopically recorded and plotted.

### Luciferase reporter assays

The 100 ng indicated luciferase reporter vectors were introduced into 1 × 10^3^ H1395 cells coupled with indicated dose of pcDNA3.1-Flag-GMFG and 10 ng pLK by Lipofectamine 3000 [18]. For another assays, the 100 ng indicated luciferase reporter vectors and 10 ng pLK were delivered into GMFG-deficient A549 cells. 48 h later, the luciferase activity was examined by Dual Luciferase Reporter Assay System. Firefly luciferase reporter activity was normalized to Renilla luciferase activity

## Tumor formation in nude mice

12 SPF nude mice aged 4-week were purchased from the Animal Resource Center at the Wuhan Institute of Virology, Chinese Academy of Sciences, China. Ethical approval was granted by Animal Ethic Committee of the local hospital, and all assays with rats were fully complied with the Declaration of Helsink. All mice were caged at a constant temperature (22–25°C) with 40–50% humidity with a 12-h: 12 h light-dark. Water and food was provided. 1 × 10^7^ GMFG-deficient A549 cells or the control cells were implanted subcutaneously into the flank of nude mice. 28 days after tumor inoculation. All mice were anesthetized and euthanized before the tumors were taken out and weighted.

### Statistical analysis

Statistical calculations were carried out with Graphpad Prism 9.0. The significant difference between two groups was determined by Student’s t test, one ANOVA was used for comparisons among multiply groups. All the data were presented as mean ± SD. P < 0.05 were considered statistically significant.

## Results

Firstly, a group of bioinformatics analysis verified the prognostic and diagnostic implication in lung cancer. Next, the loss and gain functional assays were conducted to dissect the critical role of GMFG during lung carcinomagenesis. Finally, detailed machinery was uncovered.

### GMFG expression is down-regulated in lung cancer tissues

**Figure 1.**
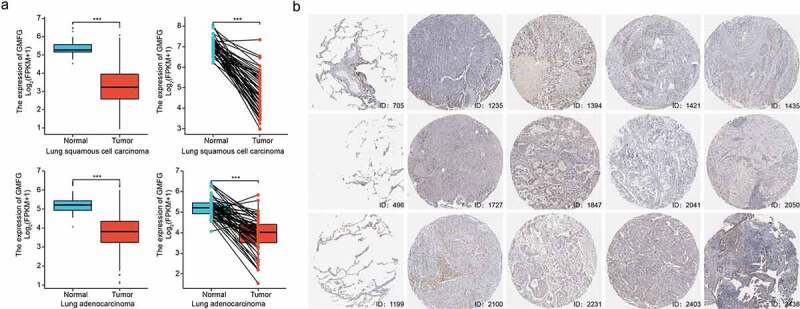
GMFG expression is down-regulated in lung cancer tissues a, The GMFG expression in LUAD/LUSC samples in TCGA database and normal lung tissues from TCGA+GTEx database. b.The GMFG expression in normal lung and LUAD/LUSC samples is shown using TCGA database. c. The results of immunohistochemistry of GMFG in normal lung and LUAD/LUSC tissues using THPA database are displayed. LUAD: lung adenocarcinoma; TCGA: The Cancer Genome Atlas; THPA: The Human Protein Atlas. GTEx:Genotype-Tissue Expression.Prognosis, diagnostic analysis of GMFG in TCGA cohort.

**Figure 2.**
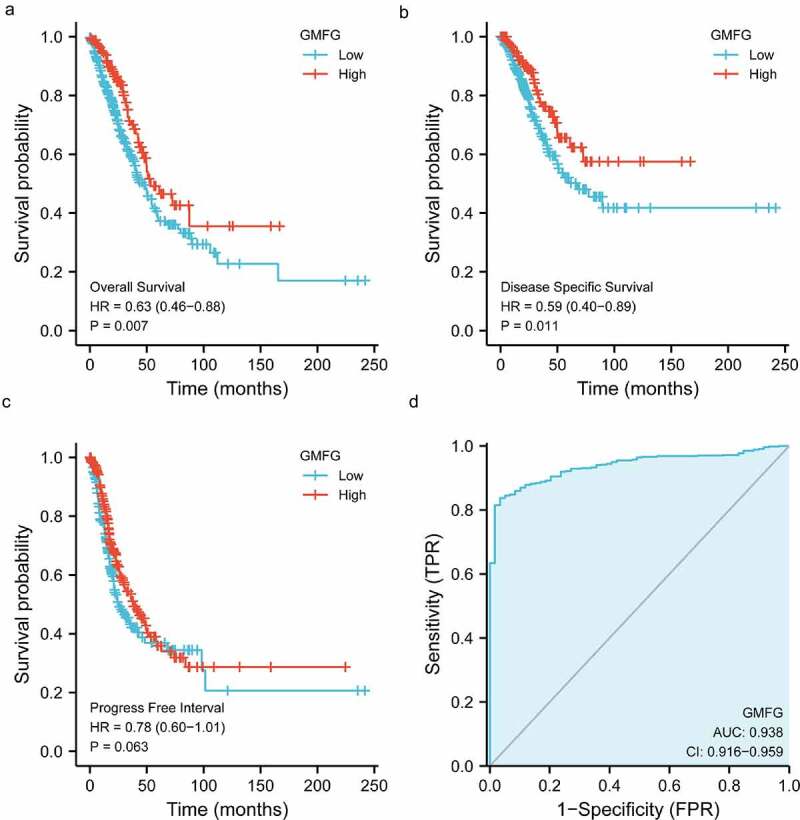
Prognosis, diagnostic analysis of GMFG in TCGA cohort a. Overall survival curves showing overall survival in GMFG high and low expression groups. b. Disease specific survival curves showing overall survival in GMFG high and low expression groups. c. Progress Free Interval curves showing overall survival in GMFG high and low expression groups. d. The diagnostic values of GMFG expression in lung cancer using ROC curve analysis.

To mine the biofunction of GMFG in lung cancer, we downloaded the cohort of Lung LUSC and LUAD from TCGA database coupled with GTEx database. As illustrated in Figure 1(a), the GMFG mRNA level was downregulated in LUAD and LUSC tissues compared with those in TCGA normal and GTEx normal lung tissues. The poor mRNA level of GMFG was also observed in TCGA tissues when compared with those in TCGA normal tissues (Figure 1(b)). The downregualted GMFG expression was further warranted in immunohistochemistry (IHC) from THPA database (Figure 1(c)). Furthermore, the correlation between GMFG mRNA level and clinical-pathological parameters of individuals suffering from LUAD and LUSC was analyzed based on the downloaded baseline information from TCGA. As illustrated in Table 1, strong associations was observed with T stage, gender and age of LUAD patients and GMFG expression. Subsequently, Kaplan Meier survival analysis were conducted to assess the prognostic value of GMFG in lung cancer based on the TCGA database. The result showed that low GMFG in lung cancer tissues was tightly linked with inferior overall survival compared with its high expression (Figure 2(a)). The shorter disease specific survival was also displayed in low-GMFG patients suffering from lung cancer compared with high-GMFG patients (Figure 2(b)), through no significance was found in progress free interval between two groups of patients (Figure 2(c)). GMFG expression also presented statistical diagnostic values in lung cancer (Figure 2(d)). Overall, these outcome indicates that GMFG might play an important role during lung cancer malignancy.

**Table 1.** Correlation between GMFG expression and clinicopathologic characteristics of LUAD patients

Characteristic	Low expression of GMFG	High expression of GMFG	p
n	267	268	
T stage, n (%)			0.024
T1	72 (13.5%)	103 (19.4%)	
T2	153 (28.8%)	136 (25.6%)	
T3	29 (5.5%)	20 (3.8%)	
T4	12 (2.3%)	7 (1.3%)	
N stage, n (%)			0.468
N0	168 (32.4%)	180 (34.7%)	
N1	47 (9.1%)	48 (9.2%)	
N2	43 (8.3%)	31 (6%)	
N3	1 (0.2%)	1 (0.2%)	
M stage, n (%)			0.439
M0	180 (46.6%)	181 (46.9%)	
M1	15 (3.9%)	10 (2.6%)	
Pathologic stage, n (%)			0.191
Stage I	134 (25.4%)	160 (30.4%)	
Stage II	66 (12.5%)	57 (10.8%)	
Stage III	47 (8.9%)	37 (7%)	
Stage IV	15 (2.8%)	11 (2.1%)	
Primary therapy outcome, n (%)			0.079
PD	45 (10.1%)	26 (5.8%)	
SD	18 (4%)	19 (4.3%)	
PR	2 (0.4%)	4 (0.9%)	
CR	157 (35.2%)	175 (39.2%)	
Gender, n (%)			0.034
Female	130 (24.3%)	156 (29.2%)	
Male	137 (25.6%)	112 (20.9%)	
Race, n (%)			0.298
Asian	5 (1.1%)	2 (0.4%)	
Black or African American	31 (6.6%)	24 (5.1%)	
White	197 (42.1%)	209 (44.7%)	
Age, n (%)			0.064
≤65	139 (26.9%)	116 (22.5%)	
>65	120 (23.3%)	141 (27.3%)	
Residual tumor, n (%)			0.465
R0	182 (48.9%)	173 (46.5%)	
R1	8 (2.2%)	5 (1.3%)	
R2	1 (0.3%)	3 (0.8%)	
Anatomic neoplasm subdivision, n (%)			0.196
Left	94 (18.1%)	111 (21.3%)	
Right	164 (31.5%)	151 (29%)	
Anatomic neoplasm subdivision2, n (%)			1.000
Central Lung	35 (18.5%)	27 (14.3%)	
Peripheral Lung	73 (38.6%)	54 (28.6%)	
number_pack_years_smoked, n (%)			0.863
<40	97 (26.3%)	91 (24.7%)	
≥40	96 (26%)	85 (23%)	
Smoker, n (%)			0.066
No	30 (5.8%)	45 (8.6%)	
Yes	233 (44.7%)	213 (40.9%)	
OS event, n (%)			0.118
Alive	162 (30.3%)	181 (33.8%)	
Dead	105 (19.6%)	87 (16.3%)	
DSS event, n (%)			0.220
Alive	182 (36.5%)	197 (39.5%)	
Dead	66 (13.2%)	54 (10.8%)	
PFI event, n (%)			0.901
Alive	153 (28.6%)	156 (29.2%)	
Dead	114 (21.3%)	112 (20.9%)	
Age, meidan (IQR)	65 (58.5, 71)	68 (59, 73)	0.033

### GMFG overexpression constrains the lung cancer cell proliferation

**Figure 3.**
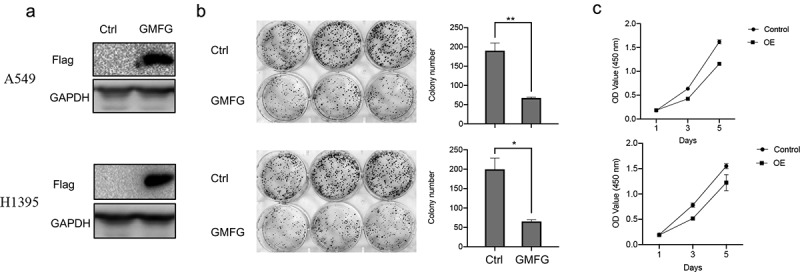
GMFG enforced expression inhibits the lung cancer cell proliferation. Overexpression of GMFG inhibited proliferation and colony formation of lung cancer cells in vitro. a. The overexpression efect of Flag-tagged GMFG-overexpressed plasmid in lung cancer cells. b. Overexpression of GPX3 inhibited colony formation of A549 and H1395 cells c.Overexpression of GMFG inhibited proliferation of A549 and H1395 cells. *P < 0.05.

To appraise the effect of GMFG in lung cancer, we firstly dissected its effect on lung cancer cell proliferation *in vitro*. The constructs expressing FLAG-tagged GMFG and the empty vectors were introduced into A549 and H1395 cells. The expression of GMFG was evidently elevated in lung cancer cells (Figure 3(a)). The lower colony formatting rate was also examined in GMFG-overexpressing lung cancer cells compared those in control groups (Figure 3(b)). Outcome of CCK8 assays demonstrated that overexpression of GMFG suppressed the proliferation of lung cancer cells (Figure 3(c)).

### GMFG deficiency accelerates the lung cancer cell proliferation

**Figure 4.**
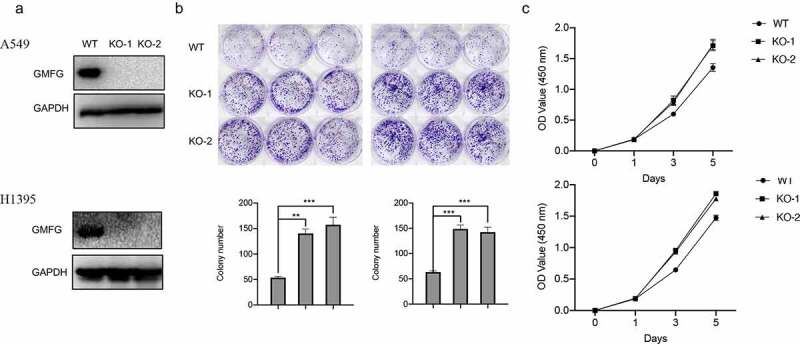
GMFG depletion accelerates the lung cancer growth in vitro and in vivo. a.The GMFG depletion was verified by western blots. b. Depletion of GMFG increased colony formation of A549 and H1395 cells. c. Depletion of GMFG promoted proliferation of A549 and H1395 cells.

We continuously assessed GMFG depletion on lung cancer cell *in vitro*. Before that, we employed CRISP/Cas9 technology to mediate GMFG knockout. The expression of endogenous GMFG was completely removed in lung cancer cells (Figure 4(a)). More interesting, GMFG knockout enhanced the number of cellular colony in lung cancer cell (Figure 4(b)). Likewise, the higher proliferative rate was also detected in GMFG-deficient lung cancer cells relative to that in normal cancer cells (Figure 4(c)). Collectively, GMFG depletion expression reduces the proliferation of lung cancer cell *in vitro*.

### GMFG regulates p53 signaling pathway

**Figure 5.**
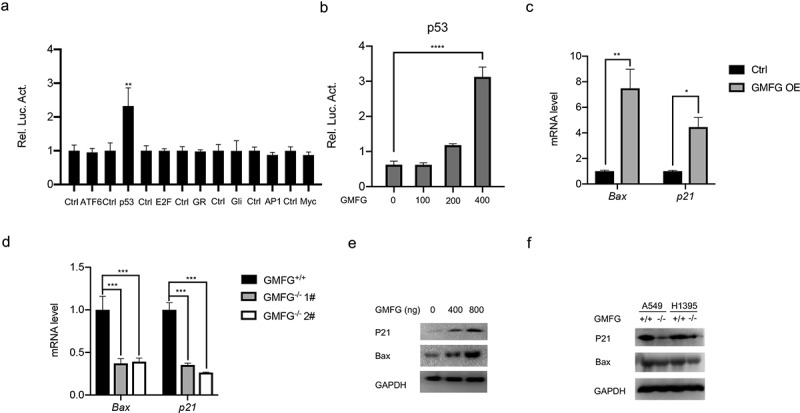
GMFG regulates p53 signaling pathway GMFG has the strongest effect on p53 transcriptional activity. a. A549 cells were cotransfected with ARE reporterfirefly luciferase (100 ng), pRL-TK (10 ng) and 50 ng GMFG-overexpressing vectors. Reporter assays were performed 24 h after transfection. b. A549 cells were cotransfected with indicated reporter firefly luciferase (100 ng), pRL-TK (10 ng) and GMFG-overexpressing vectors (0, 100, 200, 400ng). c and d. Bax and p21 expression in GMFG-overexpressing A549 cells and the control groups. e and f. Bax and p21 expression in GMFG-deficient H1395 cells and the control groups.

To investigate how GMFG manipulates the lung cancer progress, we carried out luciferase reporter assays to screen the potential signaling way GMFG regulates. We disclosed that GMFG failed to increase ATF6, E2F, GR, Gli, AP1 and Myc but evidently increased p53 transcriptional activity (Figure 5(a)). More importantly, when transfected with different dose of GMFG-overexpressing vectors into A549 cells, we also detected a dose-dependent increment of p53-driven luciferase activities (Figure 5(b)). BAX and p21 are two critical downstream effectors of p53 signaling pathway. Hence, we also detected their expression in lung cancer cells when GMFG overexpression or deficiency. As described in Figure 5(c,d), Bax and p21 mRNA levels and protein expression were elevated in A549 cells with GMFG overexpression, and yet they were reduced in GMFG-deficient H1395 cells (Figure 5(d,e)). Therefore, GMFG might drive p53 signaling pathway to inhibit lung cancer cell proliferation.

### GMFG deficiency promotes tumor growth in vivo

**Figure 6.**
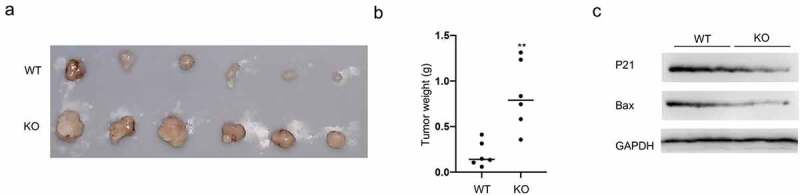
GMFG depletion promotes tumorigenesis in vivo a. Tumors were excised after euthanizing mice at the end of study, and two representative tumors from each group is shown to depict tumor size. b. Tumor weight were calculated. c. Total tissue lysates, prepared from the excised tumors, were utilized to measure the expression level of p21, Bax and GAPDH by Western blot.

Considering the *in vitro* results, we figured out the consequence of GMFG depletions *in vivo*. GMFG-deficient A549 cells were subcutaneously injected into nude mice and then the tumor size and weight were recorded. As depicted in Figure 6(a,b), upon GMFG knockout, the tumor volumes and weights were enlarged. More interesting, GMFG deficiency reduced the P21 and Bax expression (Figure 6(c)). All these data suggested that GMFG promotes lung cancer cell proliferation in vitro and favors tumorgenesis *in vivo*.

## Discussion

Identified as a critical regulator of cytokine-responsive protein, GMFG has been reportedly essential for various physiopathological processes [11]. Despite accumulating molecules clarified in cancer tumorigenesis, neither the roles of GMFG and mechanistic details remain enigmatic. This study disclosed that GMFG was downregualted in lung cancer tissues, and its expression was powerful in determining lung cancer prognosis and diagnosis. Moreover, overexpressing GMFG constrained the lung cancer cell proliferation while depleting GMFG showed an opposite scenario. More importantly, the tumor-promoting role of GMFG knockout was also validated in mice bearing A549 xenografts. Additionally, GMFG unregulated p53 signaling pathway, which inhibited lung cancer progression. Our findings suggested the essentiality of GMFG during lung cancer malignancy, and upregulating GMFG might be a promising approach for interrupting lung cancer malignancy.

Previous investigation about the expression and biofunction of GMFG in cancers has hitherto remained limited. Lan *et al* demonstrated that GMFG expression showed a contradictory effect in different neoplasms, depending on context [12]. Our findings demonstrated that GMFG was richly expressed in lung cancer based on bioinformatics analysis. Mounting evidence has demonstrated that GMFG is beneficial or detrimental in the cancer progression by regulating tumor cell behaviors. Our outcome demonstrated that ectopic GMFG expression restrained lung cancer cell proliferation. Conversely, GMFG deficiency promoted lung cancer growth *in vitro* and *in vivo*. Therefore, our data suggested that GMFG functions as a tumor-suppressor negatively regulating lung cancer.

To further ascertain how GMFG regulates lung cancer progression, we utilized a set of luciferase reporter vectors to screen the signaling interrupted by GMFG. Interesting, GMFG just increased p53 transcriptional activity in lung cancer cells. However, p53 transcriptional activity was downregulated in GMFG-deficient lung cancer cells. As a transcription factor, p53 interferences with DNA repair, cellular proliferation and senescence and thereby suppresses tumor progression [19,20]. P53 gene is verified as one of most frequently alternated anti-oncogene [21,22]. Furthermore its mutation occurs 53% patient suffering from NSCLC [23]. P21 and Bax are the core member of p53 signaling pathway in executing cell cycle arrest and apoptosis, respectively [24,25]. During the induction of apoptosis, the released p53 from the nucleus to the mitochondria interacts with p21 and leads to Bax liberation, resulting in proapoptotic or anti-proliferative abilities of tumor cells [25]. Therefore, we further examined their expression in lung cancer cells when GMFG ectopic expression and knockout. Our data showed that overexpression GMFG resulted in the elevated expression of P21 and Bax expression in lung cancer cells, while GMFG knockout produced an opposite results. Indeed, we observed obviously increased p21 and Bax expression in an A549 NSCLC xenograft tumors with GMFG knockout. Therefore, GMFG exerts its tumor-suppressive function through activating p53 signaling pathway via interacting p21 and liberating Bax. P53 activation reportedly disables lung tumor transformation and suppresses tumorgenesis. Furthermore, these findings are reminiscent of actin-related protein 2/3 (Arp2/3) complex that interplays with GMFG, governing actin dynamics during cell motility and thereby involving in several carcinogenesis [26,27]. In NSCLC, Arp2/3 complex is required for cytoskeleton assembly in lung cancer cells [8]. A transcriptional co-effector of p53 JMY is responsible for Arp2/3 activation and interfere the motility of human leukemia cells [28]. Overall, our findings suggest that GMFG might suppress lung cancer cell growth through activating p53 signaling pathway.

## Conclusion

Collectively, this investigation demonstrates the novel tumor-suppressing role of GMFG on lung cancer cell proliferation through the p53 signaling pathway. In spite of our results provides a novel potential target for lung cancer intervention, the mechanism interceding GMFG and p53 in lung cancer still requires further verification.

## References

[1] Houston T. Screening for Lung Cancer. Med Clin North Am. 2020;104(6):1037–1050. 33099449 10.1016/j.mcna.2020.08.005

[2] Suster DI, Mino-Kenudson M. Molecular Pathology of Primary Non-small Cell Lung Cancer. Arch Med Res. 2020;51(8):784–798. 32873398 10.1016/j.arcmed.2020.08.004

[3] Thai AA, Solomon BJ, Sequist LV, et al. Lung cancer. Lancet. 2021;398(10299):535–554. 34273294 10.1016/S0140-6736(21)00312-3

[4] Boczkowska M, Rebowski G, Dominguez R. Glia maturation factor (GMF) interacts with Arp2/3 complex in a nucleotide state-dependent manner. J Biol Chem. 2013;288(36):25683–25688. 23897816 10.1074/jbc.C113.493338 PMC3764776

[5] Lippert DN, Wilkins JA. Glia maturation factor gamma regulates the migration and adherence of human T lymphocytes. BMC Immunol. 2012;13(1):21. 22510515 10.1186/1471-2172-13-21 PMC3447661

[6] Zuo P, Fu Z, Tao T, et al. The expression of glia maturation factors and the effect of glia maturation factor-gamma on angiogenic sprouting in zebrafish. Exp Cell Res. 2013;319(5):707–717. 23333559 10.1016/j.yexcr.2013.01.004

[7] Ikeda K, Kundu RK, Ikeda S, et al. Glia maturation factor-gamma is preferentially expressed in microvascular endothelial and inflammatory cells and modulates actin cytoskeleton reorganization. Circ Res. 2006;99(4):424–433. 16873721 10.1161/01.RES.0000237662.23539.0b

[8] Aerbajinai W, Ghosh MC, Liu J, et al. Glia maturation factor-gamma regulates murine macrophage iron metabolism and M2 polarization through mitochondrial ROS. Blood Adv. 2019;3(8):1211–1225. 30971398 10.1182/bloodadvances.2018026070 PMC6482362

[9] Zuo P, Ma Y, Huang Y, et al. High GMFG expression correlates with poor prognosis and promotes cell migration and invasion in epithelial ovarian cancer. Gynecol Oncol. 2014;132(3):745–751. 24486602 10.1016/j.ygyno.2014.01.044

[10] Wang H, Chen Z, Chang H, et al. Expression of glia maturation factor gamma is associated with colorectal cancer metastasis and its downregulation suppresses colorectal cancer cell migration and invasion in vitro. Oncol Rep. 2017;37(2):929–936. 28075454 10.3892/or.2017.5361

[11] Yang Y, He X, Tang QQ, et al. GMFG has potential to be a novel prognostic marker and related to immune infiltrates in breast cancer. Front Oncol. 2021;11:629633. 34367945 10.3389/fonc.2021.629633 PMC8343142

[12] Lan A, Ren C, Wang X, et al. Bioinformatics and survival analysis of glia maturation factor-gamma in pan-cancers. BMC Cancer. 2021;21(1):423. 33863293 10.1186/s12885-021-08163-2 PMC8052856

[13] Tomczak K, Czerwinska P, Wiznerowicz M. The Cancer Genome Atlas (TCGA): an immeasurable source of knowledge. Contemp Oncol. 2015;19(1A):A68–77. 10.5114/wo.2014.47136 PMC4322527 25691825

[14] Wu M, Shang X, Sun Y, et al. Integrated analysis of lymphocyte infiltration-associated lncRNA for ovarian cancer via TCGA, GTEx and GEO datasets. PeerJ. 2020;8:e8961. 32419983 10.7717/peerj.8961 PMC7211406

[15] Doench JG, Fusi N, Sullender M, et al. Optimized sgRNA design to maximize activity and minimize off-target effects of CRISPR-Cas9. Nat Biotechnol. 2016;34(2):184–191. 26780180 10.1038/nbt.3437 PMC4744125

[16] Shahid S, Shahid W, Shaheen J, et al. Circulating miR-146a expression as a non-invasive predictive biomarker for acute lymphoblastic leukemia. Sci Rep. 2021;11(1):22783. 34815474 10.1038/s41598-021-02257-4 PMC8611079

[17] Zhang L, Cheng H, Yue Y, et al. H19 knockdown suppresses proliferation and induces apoptosis by regulating miR-148b/WNT/beta-catenin in ox-LDL -stimulated vascular smooth muscle cells. J Biomed Sci. 2018;25(1):11. 29415742 10.1186/s12929-018-0418-4 PMC5804091

[18] Garcia-Maquilon I, Rodriguez PL, Vaidya AS, et al. A Luciferase Reporter Assay to Identify Chemical Activators of ABA Signaling. Methods Mol Biol. 2021;2213:113–121. 33270197 10.1007/978-1-0716-0954-5_10

[19] Liu J, Zhang C, Wang J, et al. The Regulation of Ferroptosis by Tumor Suppressor p53 and its Pathway. Int J Mol Sci. 2020;21. 10.3390/ijms21218387 PMC7664917 33182266

[20] Yang FY, Zhang L, Zheng Y, et al. Dexmedetomidine attenuates ischemia and reperfusion-induced cardiomyocyte injury through p53 and forkhead box O3a (FOXO3a)/p53-upregulated modulator of apoptosis (PUMA) signaling signaling. Bioengineered. 2022;13(1):1377–1387. 34974801 10.1080/21655979.2021.2017611 PMC8805856

[21] Duffy MJ, Synnott NC, Crown J. Mutant p53 as a target for cancer treatment. Eur J Cancer. 2017;83:258–265. 28756138 10.1016/j.ejca.2017.06.023

[22] Hong B, van den Heuvel AP, Prabhu VV, et al. Targeting tumor suppressor p53 for cancer therapy: strategies, challenges and opportunities. Curr Drug Targets. 2014;15(1):80–89. 24387333 10.2174/1389450114666140106101412

[23] Zhang T, Li Y, Zhu R, et al. Transcription Factor p53 Suppresses Tumor Growth by Prompting Pyroptosis in Non-Small-Cell Lung Cancer. Oxid Med Cell Longev. 2019;2019:8746895. 31737176 10.1155/2019/8746895 PMC6815571

[24] Fan Y, Wang Y, Fu S, et al. Methylation-regulated ZNF545 inhibits growth of the p53-mutant KYSE150 cell line by inducing p21 and Bax. Exp Ther Med. 2019;18(3):1563–1570. 31410110 10.3892/etm.2019.7737 PMC6676145

[25] Kim EM, Jung CH, Kim J, et al. The p53/p21 Complex Regulates Cancer Cell Invasion and Apoptosis by Targeting Bcl-2 Family Proteins. Cancer Res. 2017;77(11):3092–3100. 28377455 10.1158/0008-5472.CAN-16-2098

[26] Molinie N, Gautreau A. The Arp2/3 Regulatory System and Its Deregulation in Cancer. Physiol Rev. 2018;98(1):215–238. 29212790 10.1152/physrev.00006.2017

[27] Rotty JD, Wu C, Bear JE. New insights into the regulation and cellular functions of the ARP2/3 complex. Nature reviews. Mol Cell Biol. 2013;14(1):7–12. 10.1038/nrm3492 23212475

[28] Zuchero JB, Coutts AS, Quinlan ME, et al. p53-cofactor JMY is a multifunctional actin nucleation factor. Nat Cell Biol. 2009;11(4):451–459. 19287377 10.1038/ncb1852 PMC2763628

